# Influence of substrate on electricity generation of *Shewanella loihica* PV-4 in microbial fuel cells

**DOI:** 10.1186/1475-2859-13-69

**Published:** 2014-05-16

**Authors:** Wenguo Wu, Fei Yang, Xing Liu, Linling Bai

**Affiliations:** 1 College of Chemical Engineering, Huaqiao University, 361021 Xiamen, P. R. China; 2 State Key Laboratory of Bioelectronics, Biological science and medical engineering department, Southeast University, 210096 Nanjing, P. R. China; 3 Key Laboratory of Environmental Medicine Engineering, Ministry of Education, School of Public Health, Southeast University, 210009 Nanjing, P. R. China

## Abstract

**Background:**

The substrate, serving as carbon and energy source, is one of the major factors affecting the performance of microbial fuel cells (MFCs). We utilized BIOLOG system to rapidly screen substrates for electricigens, and further evaluated influence of these substrates on electricity generation of *Shewanella loihica* PV-4 in MFCs.

**Results:**

Three of most favorable substrates (lactate acid, formic acid and cyclodextrin) with OD_590/750_ of 0.952, 0.880 and 0.849 as well as three of most unfavorable substrates (galactose, arabinose and glucose) with OD_590/750_ of 0.248, 0.137 and 0.119 were selected by BIOLOG system under aerobic conditions. The chronoamperometry results showed that MFCs fed with these substrates exhibited different current behaviors. Cyclic voltammograms results showed that arabinose, galactose and glucose promoted electron transfer from outer membrane *c*-Cyts of cells to the electrode surface. Lactic acid, formic acid and cyclodextrin produced lower quantity of electric charge of 10.13 C, 9.83 C and 10.10 C, the corresponding OD_600_ value was 0.180, 0.286 and 0.152 in BES; while galactose, arabinose and glucose generated higher quantity of electric charge of 12.34 C, 13.42 C and 17.45 C, and increased OD_600_ values were 0.338, 0.558 and 0.409 in BES. SEMs results showed that plenty of plump and stretched cells as well as appendages were observed when lactic acid, formic acid, and cyclodextrin were utilized as substrates, while sparse cells in short shape were obtained when galactose, arabinose and glucose were used as substrates.

**Conclusions:**

These results suggest that substrate not only has important role in electrochemical performances of MFCs but also in biological properties of electricigens. Lactic acid, formic acid, and cyclodextrin beneficial for cell growth under aerobic conditions are unfavourable for planktonic cell growth and current generation under anaerobic conditions, while consumptions of galactose, arabinose and glucose adverse to cell growth under aerobic conditions are favourable for planktonic cell growth and current generation under anaerobic conditions due to the increase of cell numbers with more outer membrane *c*-Cyts transferring electrons between the electrode surface and cells.

## Background

Microbial fuel cells (MFCs), harvesting electricity from renewable biomass, have attracted great interest in the area of wastewater treatment, bioremediation, biosensors and so on [1-4]. The microbial fuel cell consists of an anode, which accepts electrons released from the microbial metabolism and passes electrons to a cathode, where they are accepted by molecular oxygen. The knowledge that bacteria can generate electric current was first reported by Potter [5]. However, the low power density is still one of the main limiting factors restricting the practical application of MFC [6,7]. To overcome this problem, many researchers devote research to the optimization of MFC construction and operation condition [8,9], screening of active electricigens [10], as well as the modification of electrode with nanostructures [11-14]. The substrate serving as carbon and energy source is also considered as a major factor which affects the performance of MFC [15]. However, most of these studies are focused on the limited sorts of single substrate or complex substrates in MFCs with pure culture or activated sludge, respectively.

*Shewanella loihica* PV-4, a dissimilatory metal reducing bacterium isolated from the Loihi Seamount in Hawaii, has received attention because it generates higher current density than other *Shewanella* strains [16]. In contrast to most *Shewanella* species, *S. loihica* PV-4 was able to utilize fumarate, galactose, glucose, citrate, lactate, malate, maltose, N-acetylglucosamine, succinate and alanine as substrates but unable to utilize acetate, propionate or Tween 40 [17]. Although the effect of lactate [16] and a mix of volatile fatty acids [18] as substrates on the performance of MFCs have been evaluated, there is rare report about influence of other single substrates on electricity generation performance and cell growth as well as the interaction between them.

BIOLOG system, taking advantage of microbe’s ability to use particular carbon sources to produce a unique “fingerprint” of 95 single carbon sources in a MicroPlate, is widely used for the assessment of bacterial functional diversity in environmental samples [18-20]. The ability of a microbe to use a particular carbon source produces respiration, which reduces a tetrazolium redox dye and causes a color change in that well. The end result is a pattern of colored wells that is characteristic for that organism. Herein, we utilize BIOLOG system to rapidly screen favorable and unfavorable substrates for the growth of *S. loihica* PV-4, and further evaluate influence of these substrates on electricity generation of *S. loihica* PV-4 in MFCs.

## Results and discussion

### Substrate screening results

**Figure 1:**
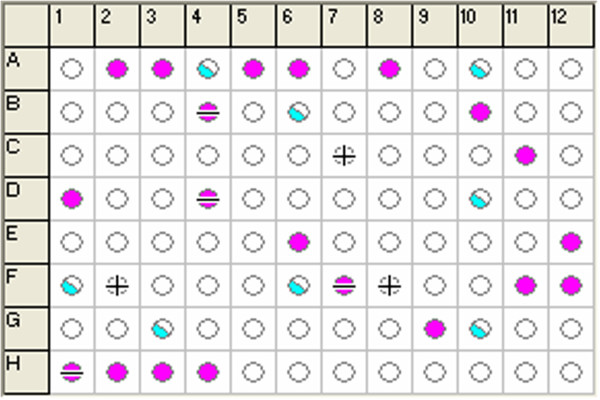
**Profiling of *S. loihica *PV-4 cultures in GN2 MicroPlate using MicroLog software after 25 h of culture.** Purple color: positive, no color: negative, plus and minus: mismatches.

The substrate metabolism process of *S. loihica* PV-4 was indicated by a tetrazolium redox dye in GN2 MicroPlate. The specific pattern of color change on the plate provided an identifiable metabolic fringerprint. As shown in Figure 1, some wells exhibited noticeable purple color in comparison with the control well (A1) with water as substrate after 25 h of aerobic culture. Most of negative wells showed no obvious color, it was indicated that the substrates in them are favorable for cellular growth and respiration. In addition, some wells exhibited half cyan color, it was suggested that the substrates in them are unfavorable. Combined with these OD_590/750_ results, we chose three of the most favorable and unfavorable substrates to study substrate influence on electricity generation of *S. loihica* PV-4 in MFCs. They were D, L-lactic acid (E6, OD_590/750_ = 0.952), formic acid (D4, OD_590/750_ = 0.880), α-cyclodextrin (A2, OD_590/750_ = 0.849), D-galactose (B4, OD_590/750_ = 0.248), L-arabinose (A10, OD_590/750_ = 0.137) and α-D-glucose (B6, OD_590/750_ = 0.119).

### Chronoamperometry

**Figure 2:**
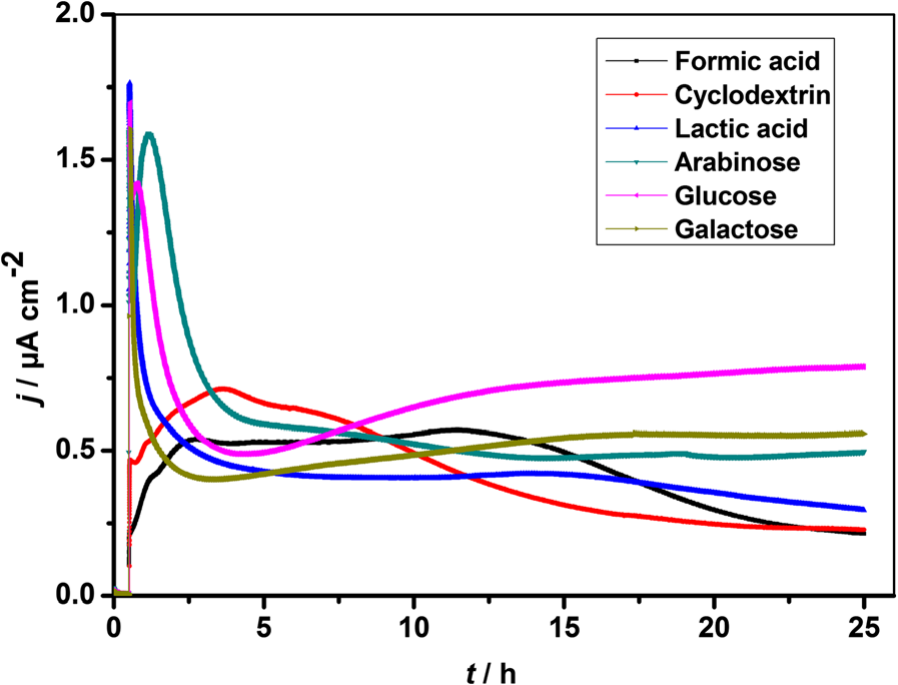
**Substrate effect on the electricity generation of *S. loihica *** **PV-4 in MFCs.**

The electricity generation results of *S. loihica* PV-4 in bioelectrochemical system (BES) at a poised potential of 0.2 V for 25 h with lactic acid, formic acid, cyclodextrin, galactose, arabinose and glucose as substrates were shown in Figure 2. With the addition of cells, an oxidative current was generated on ITO electrode, whereas no redox response was observed on the electrode without the addition of cells (data not shown). The oxidative current is ascribed to the electrical connection from outer membrane *c*-Cyts of cells to the electrode [21-24]. As shown in Figure 2, the BES fed with different substrates in the presence of cells exhibited different current behaviors. When formate acid and cyclodextrin were used as substrates, an oxidative current of 0.19 μA cm^−2^ and 0.46 μA cm^−2^ was generated, and gradually grew with a broad current peak of 0.71 μA cm^−2^ and 0.57 μA cm^−2^ until ~11.4 h and ~3.2 h, then decreased slowly to a final current of 0.21 μA cm^−2^ and 0.23 μA cm^−2^ respectively. When lactic acid, galactose, arabinose and glucose were used as substrates, an immediate current peak of 1.76 μA cm^−2^, 1.49 μA cm^−2^, 1.58 μA cm^−2^ and 1.69 μA cm^−2^ was generated and gradually decreased or increased to a final current of 0.30 μA cm^−2^, 0.56 μA cm^−2^, 0.50 μA cm^−2^ and 0.79 μA cm^−2^ respectively. Similar chronoamperometry results were observed in the single-chamber, three-electrode electrochemical system with ITO as working electrode and *S. loihica* PV-4 as electricigens [22,25]. However, current was greatly enhanced on graphite (5 μA cm^−2^) [25], nanograss array boron-doped diamond (1.2 μA cm^−2^) [13], and ITO coated with polyaniline nanowire network electrodes (45 μA cm^−2^) [26] due to high surface roughness and nanostructured surface. It was reported that an important aspect of achieving high power density was having a low internal resistance [27]. The total internal resistance consists of anodic, membrane, cathodic, and electrolyte resistance [28]. Among these, anodic resistance is the main limiting factor in internal resistance in this single-chamber, three-electrode electrochemical system where the cathode is platinum wire. The low current generated on ITO electrode was ascribed to increase of anodic resistance from small population of bacteria attached to the plane electrode surface [27]. However, ITO is widely used for spectroelectrochemical characterization of purified redox proteins and whole microbial cells due to its excellent optoelectronics properties [22,25]. Herein, it was chosen for the study of substrate effect on biological and electrochemical properties of *S. loihica* PV-4.

### Cylic voltammetry

**Figure 3:**
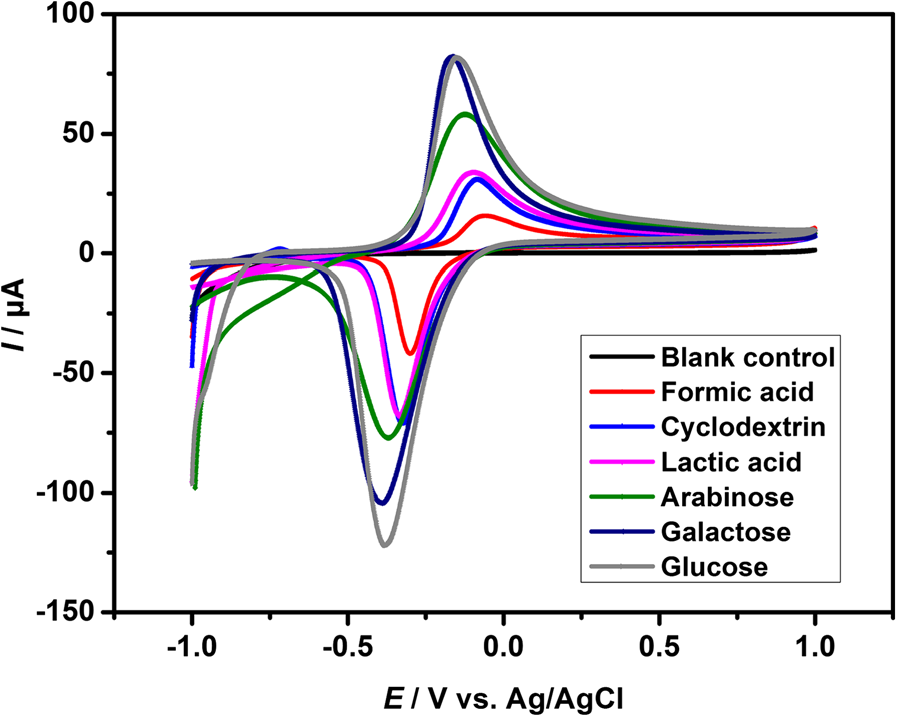
**CVs of *S. loihica *PV- 4 obtained on ITO electrode in the absence (blank control) and presence of substrates.** Scan rate: 0.01 mV s^−1^.

The effect of substrates on electron transfer between *S. loihica* PV-4 cells and ITO electrode was evaluated by cyclic voltammograms (CVs), after polarizing the electrode for 25 h. As shown in Figure 3, there were no obvious redox waves on ITO electrode in the absence of substrate. After the addition of substrate, pairs of well-defined redox waves were observed on the electrode. The current generation was ascribed to the oxidation of organic compounds coupled to reduction of electron acceptors by cells. For formic acid, cyclodextrin and lactic acid, CVs showed sharp reductive peaks at −0.298 V, −0.318 V, −0.332 V, and broad oxidative peaks at −0.060 V, −0.082 V, −0.094 V. For arabinose, galactose and glucose, quasi-reversible CVs with reductive peaks at −0.336 V, −0.338 V, −0.378 V and oxidative peaks at −0.124 V, −0.168 V, −0.124 V were exhibited on the electrode. These CVs results confirm an outer membrane *c*-Cyts-mediated electron transfer to the electrode surface [13,22]. The cells inoculated in the presence of arabinose, galactose and glucose exhibited higher redox peak currents as compared to those using formic acid, cyclodextrin and lactic acid as substrates. It was indicated that arabinose, galactose and glucose promoted electron transfer from outer membrane *c*-Cyts of cells to the electrode surface and cells fed with these substrates had superior electrochemical performances. These CVs results were in accordance with the chronoamperometry results.

### Quantity of electric charge and cell growth

**Figure 4:**
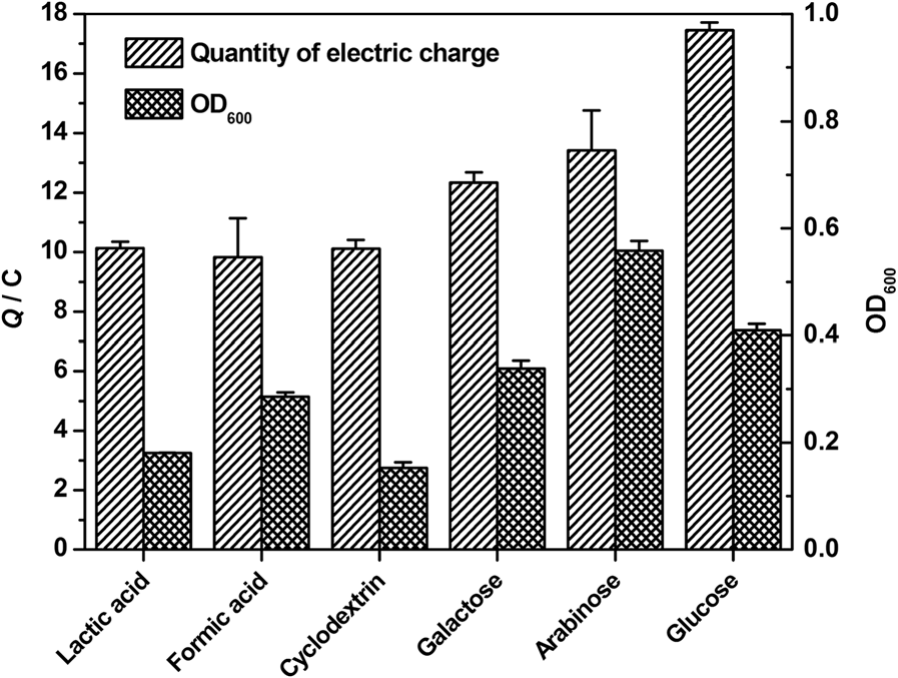
**Substrate effect on quality of electric charge and cell growth of *S. loihica *** **PV-4 in MFCs after 25 h of culture.**

To further explore the substrate effect on electricity generation, the total quantity of electric charge (*Q*) was calculated by integrating each *I-T* curve with respect to time. The formula was $Q=\int_{0}^{t}\mathrm{Idt}$[29,30]. The interrelationship between total electric charge and planktonic cell growth under anaerobic conditions with lactic acid, formic acid, cyclodextrin, galactose, arabinose and glucose as substrates in BES after 25 h was shown in Figure 4. When lactic acid, formic acid and cyclodextrin were used as substrates, the quantity of electric charge of MFC in 25 h was 10.13 C, 9.83 C and 10.10 C respectively; the corresponding OD_600_ value representing planktonic cell growth was 0.180, 0.286 and 0.152. In comparison, when galactose, arabinose and glucose were used as substrates, increased quantity of electric charge of 12.34 C, 13.42 C and 17.45 C, and increased OD_600_ values of 0.338, 0.558 and 0.409 were observed. It was suggested that lactic acid, formic acid and cyclodextrin beneficial for cell growth of *S. loihica* PV-4 under aerobic conditions were unfavorable for electricity generation and planktonic cell growth under anaerobic conditions, while galactose, arabinose and glucose adverse for cell growth under aerobic conditions were favorable for electricity generation and planktonic cell growth under anaerobic conditions. These results were in accordance with the above CVs results that BES fed with galactose, arabinose and glucose had superior electrochemical performances. This was ascribed to the increase of cell numbers with more outer membrane *c*-Cyts transferring electrons between the electrode surface and cells.

## SEM results

**Figure 5:**
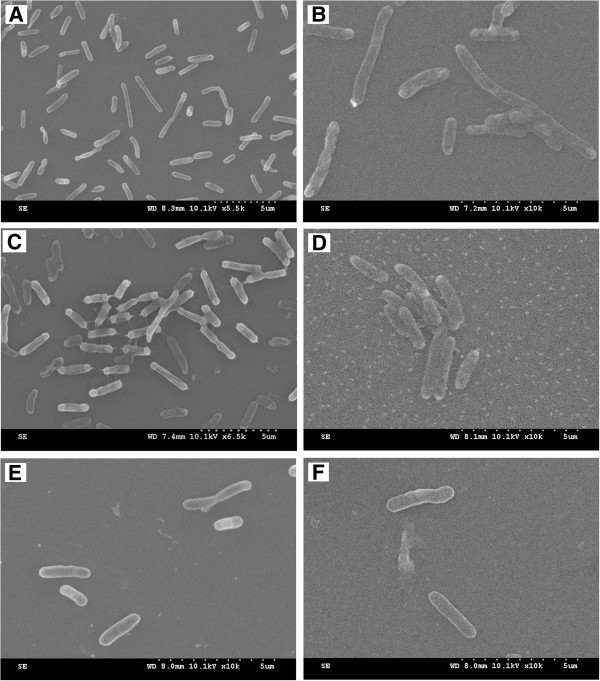
**SEM images of *S. loihica *** **PV-4 on ITO electrode in MFCs after 25 h of electricity generation with lactic acid (A), formic acid (B), cyclodextrin (C), galactose (D), arabinose (E) and glucose (F) as substrates.**

SEM images of *S. loihica* PV-4 cells on ITO electrode in MFCs after 25 h of current generation with lactic acid, formic acid, cyclodextrin, galactose, arabinose and glucose as substrates were shown in Figure 5. When lactic acid, formic acid and cyclodextrin were used as substrates, a significant amount of cells in plump and long rod shape were observed on ITO electrodes. Especially when formic acid was utilized as substrate, the length of cells nearly arrived at ~5 μm (Figure 5B). Interestingly, when cyclodextrin was used as substrate, there were obvious appendages connecting cells between each other to form a bacterial network. Similar phenomenon was observed when *Pelotomaculum thermopropionicum* was grown in monocultures on fumarate and in cocultures with *Methanothermobacter thernoautotropicus* on propionate [31]. These experimental results proved that *S. loihica* PV-4 could also produce pilus-like appendages in response to different substrates fed into MFCs. It was reported that microbial nanowires performed under unnatural conditions [32]. The OD_600_ value results mentioned above had shown that cyclodextrin was the most unfavourable substrate for planktonic cell growth under anaerobic condition. This unsuitable culture condition could have an important role in the formation of appendages. However, further researches such as the formation mechanism and electrical properties of these appendages are still necessary. When galactose, arabinose and glucose were used as substrates, the cells attached on the electrodes were very sparse and in shorter shape, indicating that the catalytic activity of each individual cell was high. In contrast, bacterial biofilm in uniform morphology was observed on the anode surface of a mixed culture MFC, operated with a high strength wastewater [33]. It was suggested that cellular morphologies together with biological and electrochemical activities of electricigens were significantly affected by substrates.

## Conclusions

Lactic acid, formic acid, cyclodextrin, galactose, arabinose and glucose serving as electron donors for *S. loihica* PV-4 in MFCs were selected by BIOLOG system. Lactic acid, formic acid and cyclodextrin beneficial for cell growth under aerobic conditions were unfavourable for planktonic cell growth under anaerobic conditions and produced lower quantity of electric charge, while galactose, arabinose and glucose adverse to cell growth were favourable for planktonic cell growth under anaerobic conditions and generated higher quantity of electric charge. The electron donor played an important role not only in electrochemical performances of cells but also in cellular morphologies, especially the formation of appendages. Further researches including underlying formation mechanism and electrical properties of appendages are still necessary.

## Methods

### Bacteria culture

*Shewanella loihica* PV-4 strain (ATCC BAA-1088) was aerobically cultured in 10 mL of Marine Broth (20 g L^−1^) at 30°C for 24 h. After centrifugation, the Marine Broth was replaced with 10 mL of defined media (DM) [21] at 30°C for 48 h with different substrates. The suspension was centrifuged for 10 min and the resultant cell suspension was washed with DM three times prior to being used for electrochemical experiments. As substrates, lactate acid, formic acid, cyclodextrin, galactose, arabinose and glucose (10 mM) were used respectively.

### Screening of substrates by BIOLOG system

*S. loihica* PV-4 was inoculated and aerobically cultured in Marine Agar (20 g L^−1^) at 30°C for 16–24 h. A uniform suspension within the specified turbidity range was prepared by dipping the swab picking up cells into the inoculating fluid. Inoculate cells (150 μl per well) into GN2 MicroPlate (Biolog Catalog #1011), incubate aerobically at 30°C for 25 h and then read by BIOLOG MicroLog System (Release 4.2, Biolog Inc., USA) with two appropriate filters (590 and 750 nm).

### Electrochemical measurements

A single-chamber, three-electrode system was used for the electrochemical measurements, where ITO electrode was used as the working electrode on the bottom of the cell [21], a platinum wire as the counter, and an Ag/AgCl (saturated KCl) electrode as the reference. The reactor filled with 4 mL DM containing 10 mM different substrates mentioned above was deaerated by purging with N_2_ gas (30 min) and subsequently injected with bacterial culture (OD_600_ 2.0) as described above at a constant poised potential of 0.2 V using a CHI 660D potentiostat (CH Instruments, Chenhua Co. Shanghai, China) at 25°C, pH 7.8. After these electrochemical measurements, the final optical density (OD_600_) of the bacterial culture in the reactor after 25 h was measured using a spectrophotometer (Mapada, China). All these experimental tests were repeated for one time.

### SEM observation

*S. loihica* PV-4 attached on the electrodes were imaged using an SEM (HITACHI, S-300 N). Samples were fixed in 2.5% glutaraldehyde for 2 h, rinsed three times in phosphate buffer (pH 7.0, 50 mM), dehydrated by alcoholic series (60, 70, 80, 90, 95, and 100%), and then air-dried.

## Competing interests

The authors declare that they have no competing interests.

## Authors’ contributions

WW carried out the electrochemical measurements, participated in the screening of substrates by BIOLOG system and drafted the manuscript. FY carried out the screening of substrates by BIOLOG system. XL participated in the electrochemical measurements and SEM observation. LB participated in its design and coordination and helped to draft the manuscript. All authors read and approved the final manuscript.
